# Impact of Mindfulness-Based Teacher Training on MBSR Participant Well-Being Outcomes and Course Satisfaction

**DOI:** 10.1007/s12671-017-0750-x

**Published:** 2017-06-06

**Authors:** Pauline Eva Ruijgrok-Lupton, Rebecca S. Crane, Dusana Dorjee

**Affiliations:** Centre for Mindfulness Research and Practice, School of Psychology, Bangor University, Bangor, LL57 2AS UK

**Keywords:** MBSR/MBCT teachers, Mindfulness-based teacher training, Mindfulness-based stress reduction, MBSR, Participant well-being outcomes, Mindfulness-based programs

## Abstract

Growing interest in mindfulness-based programs (MBPs) has resulted in increased demand for MBP teachers, raising questions around safeguarding teaching standards. Training literature emphasises the need for appropriate training and meditation experience, yet studies into impact of such variables on participant outcomes are scarce, requiring further investigation. This feasibility pilot study hypothesised that participant outcomes would relate to teachers’ mindfulness-based teacher training levels and mindfulness-based teaching and meditation experience. Teachers (*n* = 9) with different MBP training levels delivering mindfulness-based stress reduction (MBSR) courses to the general public were recruited together with their course participants (*n* = 31). A teacher survey collected data on their mindfulness-based teacher training, other professional training and relevant experience. Longitudinal evaluations using online questionnaires measured participant mindfulness and well-being before and after MBSR and participant course satisfaction. Course attendees’ gains after the MBSR courses were correlated with teacher training and experience. Gains in well-being and reductions in perceived stress were significantly larger for the participant cohort taught by teachers who had completed an additional year of mindfulness-based teacher training and assessment. No correlation was found between course participants’ outcomes and their teacher’s mindfulness-based teaching and meditation experience. Our results support the hypothesis that higher mindfulness-based teacher training levels are possibly linked to more positive participant outcomes, with implications for training in MBPs. These initial findings highlight the need for further research on mindfulness-based teacher training and course participant outcomes with larger participant samples.

## Introduction

Mindfulness-based stress reduction (MBSR) (Kabat-Zinn 2005) was developed as a mainstream, accessible vehicle for training participants in mindfulness practice and its application to chronic pain management and other life challenges (Kabat-Zinn et al. 1985). Since the introduction of MBSR, the field of mindfulness-based programs (MBPs) has developed exponentially, both in diversity of application, including mindfulness-based cognitive therapy (MBCT) for preventing depression relapse, and in empirical evidence of clinical efficacy (Cullen 2011; Khoury et al. 2013; Kuyken et al. 2016), resulting in burgeoning public interest. Accordingly, demand for mindfulness-based programs and teachers has been growing substantially, raising the issue of intervention fidelity. The essential intentions of MBSR as a way of delivering mindfulness-based teaching in mainstream settings are safeguarded through adherence to the curriculum, in terms of length and number of course sessions, course content, and home practice (Blacker et al. 2015; Dobkin et al. 2014), and embodied through authentic delivery by adequately trained teachers grounded in the practice (Kabat-Zinn 2011). Kabat-Zinn (2011) stated that “the quality of MBSR as an intervention is only as good as the MBSR instructor and his or her understanding of what is required to deliver a truly mindfulness-based program” (p. 281). However, pressing demand for more MBP teachers can conflict with the requirement for in-depth teacher training and meditation experience, widely accepted within the MBP training community as fundamental to ensuring MBPs are conveyed correctly and efficaciously (Brandsma 2017; Kabat-Zinn and Santorelli n.d.; McCown et al. 2010; Piet et al. 2016; Santorelli 2004; UK Network for Mindfulness-Based Teacher Training Organisations 2011).

Within the field of MBP pedagogy, Dobkin and Hassed (2016) provided an overview of the required teaching skills and training routes, and McCown offered a model of the ethical space emerging within MBP teaching situations as a way forward to securing teacher quality (McCown 2013). The McCown model focused on the relational aspect of the gathering of teacher and participants and on developing the relational skills of the teacher. To help safeguard teaching standards, Crane et al. (2010) identified key elements for teacher competence and training phases and developed and validated an assessment tool for teaching competence, the structure of which was adapted from the cognitive therapy adherence and competence scale (Blackburn et al. 2001): the Mindfulness-Based Intervention—Teaching Assessment Criteria (MBI-TAC) (Crane et al. 2012; Crane et al. 2013). The MBI-TAC examines different aspects of teaching, e.g. embodiment, relational skills, interactive teaching and group holding (Crane et al. 2016). The process of inquiry in MBP teaching was also investigated in depth by Crane et al. (2014). Similar central themes in the role of the mindfulness teacher were recognised by Van Aalderen et al. (2014) in their triangulated qualitative analysis of the MBCT teacher-participant relationship affecting impact on participants, namely teacher embodiment of mindfulness, empowerment of participants, teacher non-reactivity and group support. The MBI-TAC is used to support teacher development and assessment in British university-based teacher training programs and other training programs (e.g. Marx et al. 2015) and is being implemented in training programs across Europe and North America.

Notwithstanding these pioneering and pivotal advances in the spheres of training stages and competency assessment, little research has been conducted to support the assumed importance of mindfulness-based teacher training and meditation experience for participant outcomes (Crane et al. 2010; Piet et al. 2016). Van Aalderen et al. (2014) made a significant contribution to greater understanding of the role of the teacher within MBPs, yet no information was available on teachers’ qualifications, ruling out the possibility of linking their findings to teacher training and experience. Indeed, in their meta-analysis of MBPs, Khoury et al. (2013) pointed to the need for more research into the moderating effect of MBP teachers, training and experience on clinical outcomes for participants. This point was also recently highlighted by Dimidjian and Segal (2015) in the context of the six-stage development model for clinical implementation research of the National Institutes of Health (NIH) (Onken et al. 2014). Specifically, Dimidjian and Segal (2015) marked out “the thorny question of clinician training” as an essential element for future MBP implementation (p. 605), recognising the unusual requirement of professional training combined with personal practice. From a broader implementation perspective and in view of the dramatic proliferation of MBPs and teacher training programs, the UK parliamentary enquiry into the role of mindfulness in the public sector (UK Mindfulness All-Party Parliamentary Group (MAPPG) 2015) raised concerns about poorly qualified teachers offering mindfulness-based courses and considered teacher training as essential. However, without evidence linking mindfulness-based teacher training or competence to participant outcome, it is difficult to support this requirement.

Whilst to date the issue of teacher training and outcomes has not been looked at thoroughly in the MBP context, there are precedents for considering this aspect in the field of Cognitive Therapy, which informed the framework for MBI-TAC. Studies in cognitive therapy (CT) and cognitive behavioural therapy (CBT) demonstrated significant correlation between therapist training, competence and participant outcomes (e.g. Brosan et al. 2007; Milne et al. 1999). For example, Milne et al. (1999) found that advanced CT training was related to higher therapist competence and significantly improved coping strategies in patients. However, as there are considerable differences between these interventions (e.g. CT aims to modify cognitive processes, MBPs aim to change the relationship to cognitive processes), CT and MBPs cannot be directly compared. The evidence from CT regarding correlations between training and outcome is therefore not directly transferable but highlights the need for evidence in the MBP context regarding links between teacher qualifications and participant outcomes.

Based on the CT findings, we might expect similar positive relationships between participant outcome improvements and MBP teachers’ competence and levels of training and meditation experience, with implications for teacher training standards and implementation of MBPs. Whilst competence is expected to be important in MBP teacher impact, a recent study into the relationship between teacher competence and MBCT treatment outcome for depression found no significant association (Huijbers et al. 2017). Examining the relationship between teacher competence and course participant outcomes may be complicated by the process of assessing the competence of MBP teachers, which is time-consuming and requires highly trained assessors to obtain grading consistency. Hence, levels of training within an established mindfulness-based teacher training program might be more easily quantifiable and could provide a suitable starting point for initial explorations of possible links between course participant outcomes and MBP teacher qualifications, rather than teacher competence. Therefore, the purpose of this feasibility pilot study was to compare well-being outcomes of three groups of MBSR participants on courses taught by MBSR/MBCT teachers with respectively 1, 2 and 3 years of MBP teacher training at an established postgraduate mindfulness-based teacher training program. Research data were collected from MBSR/MBCT teachers on training, experience and meditation practice and from course participants at pre- and post-test stage on a range of well-being measures, including mindfulness. As no consensus exists on construct and operationalisation of mindfulness and how to best assess it (Chiesa 2013; Grossman and Van Dam 2011), we focused on participant well-being outcomes and participant satisfaction. Considering the emphasis on importance of training within MBP training literature and previous evidence regarding the impact of facilitator training in therapeutic interventions, it was anticipated that MBSR course participants taught by teachers with more advanced levels of mindfulness-based teacher training would show higher gains in well-being outcomes than participants following courses by teachers with less training. Possible relationship between course participant outcomes and MBP teaching and meditation experience of teachers, as well as other relevant professional experience, was also explored.

## Method

### Participants

The research study was approved by the ethics committee of the university where the participating postgraduate program is located, prior to participant recruitment. Participating teachers were current or former students of a postgraduate mindfulness-based teacher training program, who had completed 1, 2 or 3 years of teacher training levels on this program labelled, respectively, L1, L2 and L3. These three labels correspond broadly with Basic Teacher Training (L1), Advanced Teacher Training (L2) and Continuing Professional Development (L3) MBSR/MBCT teacher training stages identified by Crane et al. (2010). Learning in these consecutive year-long academic modules is incremental in course content, assessment and teaching requirements. Modules are assessed by a combination of written assignments integrating theory and practice and MBI-TAC assessment of teaching skills, respectively, a guided mindfulness practice (L1), a didactic course element (L2) or a selection from a complete video-recorded 8-week mindfulness-based course (L3). For the purpose of this study, the labels L1, L2 and L3 refer to the three cohorts of participating teachers and their MBSR course participants who volunteered for the study, according to the corresponding teachers’ mindfulness-based teacher training level.

The only inclusion criterion for MBSR/MBCT teachers was to have completed one or more of the three teacher training levels and to be teaching MBSR courses during the data collection period of the study. Recruited teachers invited their MBSR course attendees to participate in the research using the standard recruitment materials and protocols provided by the researcher. The MBSR courses were delivered to a general non-clinical population and adhered to the MBSR curriculum (Blacker et al. 2015). Participation of teachers and course participants was voluntary. Eleven MBSR/MBCT teachers participated in the study, two in L1 (0 males), four in L2 (1 male) and five in the L3 cohort (2 males), delivering a total of 16 MBSR courses. MBP teaching experience varied from 3 to over 100 courses, and all but one teacher (from the L2 cohort) had prior clinical or educational experience or qualifications (see Table 4). Overall MBSR course participant sample was *N* = 52 divided between the teacher levels: *n* = 2 in L1, *n* = 13 in L2 and *n* = 37 in L3. Only data from course participants who completed both pre- and post-test questionnaires were included; measurements from participants attending fewer than five out of eight teaching sessions or who started or discontinued any form of mental health treatment or therapy during their course were excluded. This resulted in final *N* = 33, *n* = 2 for L1, *n* = 9 for L2 and *n* = 22 for L3 (see Table 1). Since the five teachers in L3 taught a total of 10 courses included in the study, the average number of course participants taking part in the study was the same for each cohort, i.e. 2.2 participants per course.

**Table 1:** Overview of participating MBSR/MBCT teachers, courses and MBSR course participants in L1, L2 and L3 groups after exclusions

	L1 Group	L2 Group	L3 Group
	*n*	*n*	*n*
MBSR/MBCT teachers	2	4	5
Number of courses	2	4	10
MBSR course participants participating in study	2	9	22^a^

*L1 Group* cohort of MBSR courses taught by teachers with 1 year of MBP teacher training, *L2 Group* cohort of courses taught by teachers with 2 years of teacher training, *L3 Group* cohort of courses taught by teachers with 3 years of teacher training

^a^Recruited from a total of 10 courses taught by the five L3 teachers

### Procedure

The study followed a non-randomised controlled pre-post design. It aimed to compare mindfulness and well-being outcomes of MBSR course participants across three teacher groups, with groups differing in their levels of teacher training (1, 2 or 3 years, respectively). Each of the three groups consisted of MBSR/MBCT teachers with the same level of teacher training together with their respective MBSR course participants, enabling comparisons between cohorts. Accordingly, data were collected from two types of related study participants within each of the three cohorts, i.e. MBSR/MBCT teachers and their respective MBSR course participants. Participant descriptions are given below, and an overview of the three cohorts is provided in Table 1.

MBSR/MBCT teachers were recruited through e-mails sent by the administrator of the postgraduate mindfulness-based teacher training program to the students on the program and through personal contacts of the first author. After providing informed consent, MBSR/MBCT teachers received sample e-mails for inviting their MBSR course participants to take part in the study. To avoid bias, no details were given to teachers about the content of course participant questionnaires or to course participants about training level of their teacher. Interested participants contacted the researcher and were provided with information about the study for informed consent. Questionnaire links to a secure custom-made online data collection interface were sent out to all course participants as soon as they provided their informed consent. In line with ethical procedures and guidelines, participants had the option not to respond to questions on the questionnaires they did not want to answer. Pre-test questionnaires were completed within 1 week before the start of the MBSR courses, apart from six participants completing questionnaires up to 3 weeks beforehand. Participants who completed the online questionnaires after the start of the course were excluded. At the post-test, questionnaires were sent out the day after course ending and filled in within 1 week, except in the case of three participants experiencing difficulty accessing the online links, who completed the questionnaires within 2 weeks. Where necessary, the researcher sent reminders to participants to complete the questionnaires.

### Measures

Data from MBSR/MBCT teachers were collected pre-test with a custom-made questionnaire, which gathered information about teaching training and experience, meditation experience and other relevant professional experience in addition to age and gender. The questionnaire also asked for information about the MBSR course the teachers were teaching to assess that it adhered to the standard form and process of MBSR (Blacker et al. 2015), i.e. consisting of eight 2–2.5-h sessions and including the body scan, mindful movement and sitting meditation, with formal home practice of 30–45 min daily for course participants. Teachers were allocated to three training cohorts for analysing between-group differences for teacher variables and course participant outcomes.

Measures for course participants comprised standardised self-report questionnaires shown sensitive to well-being enhancements after MBSR training in previous studies, as well as self-report questionnaires on basic demographics and course satisfaction designed for the study. Specifically, the following measures were used: mindfulness was measured with the Five Facet Mindfulness Questionnaire (FFMQ) (Baer et al. 2006; Baer et al. 2008). FFMQ consists of 39 questions and measured facets are observing, describing, acting with awareness, non-judging and non-reactivity, deemed to represent the concept of mindfulness as understood in MBPs (Baer et al. 2006). FFMQ has been found to be effective in demonstrating significant improvements in mindfulness facets after completing an MBSR program (Carmody and Baer 2008; Vøllestad et al. 2011). Validity and reliability of FFMQ are very high, with a Cronbach’s alpha coefficient for the facets ranging between 0.78 and 0.91 (sample size of 376) (Bohlmeijer et al. 2011) and between 0.69 and 0.90 (sample size 140) (Veehof et al. 2011). In the current study, Cronbach’s *α* value at pre-test was .935, and at post-test *α* = .956.

To assess changes in self-compassion, the Self-Compassion Scale—Short Form (SCS-SF) (Raes et al. 2011) was used. It consists of 12 items measuring self-judgment, self-kindness, isolation, mindfulness, over-identification and common humanity (Raes et al. 2011). Shapiro et al. (2005) observed significant improvements in self-compassion in healthcare professionals following an MBSR program using the Self-Compassion Scale. This finding was echoed by Birnie et al. (2010) in their study with general public participants. The Long Form of the Self-Compassion Scale has a reliability value of 0.93 (Baer et al. 2006), and the short form (SCS-SF) was shown to have a reliability of 0.71 (Raes 2011). For this study, Cronbach’s value for baseline scores was *α* = .820, and at post-test *α* = .812.

To evaluate specific changes in well-being, W.H.O. (Five) Well-Being Questionnaire (WBI-5) (Bech 1998; Primack 2003) was used. This measure contains five positively framed questions, regarding energy, mood and general interest. A feasibility study on MBCT for primary care patients resulted in significant increases in WBI-5 scores (Radford et al. 2012), similar to significant increases in WBI-5 values measured in a randomised controlled trial on well-being for breast cancer patients following an MBSR program (Hoffman et al. 2012). Research documented Cronbach’s *α* value of 0.91 in a sample size of 501 patients (Löwe et al. 2004). For the current study, Cronbach’s *α* values were *α* = .878 at pre-test, and *α* = .905 at post-test.

Expected reductions in stress were measured with the Perceived Stress Scale—10 Item (PSS) (Cohen et al. 1983; Fliege et al. 2005), which contains 10 questions. Shapiro et al. (2005) noted significant decreases in perceived stress after intervention with MBSR, as did Carmody and Baer (2008). Research has demonstrated reliability values of 0.84, 0.85 and 0.86 in samples of, respectively, 64, 114 and 332 healthy college students (Cohen et al. 1983). The Cronbach’s value for baseline scores in this study was *α* = .887, and at post-test *α* = .869.

The first self-report questionnaire specifically designed for this study was a demographics survey which consisted of questions about age, gender, education, occupation and previous MBP course participation. The second self-report survey specific to this study measured course satisfaction and contained 15 questions evaluated on a 5-point Likert scale. The questions assessed aim fulfilment (“To what extent have your aims/intentions/wishes for the 8-week course been fulfilled?”), course impact on daily life (“How helpful has the course been for how you handle stress/difficulties/pain; your relationship with others; your daily activities”), amount of practice on completion of the course (“How much do you practice mindfulness now?”) and support and inspiration from group and teacher (“How helpful did you find big/small group discussions; teaching sessions; teacher support; learning in the group; the Day of Mindfulness” and “How much do you feel your teacher has helped you to understand what Mindfulness is about”, “How much do you feel the group has helped you to understand what Mindfulness is about”, “How much do you feel the teacher has inspired you to do the Home Practice”, and “How much do you feel the group has inspired you to do the Home Practice”). The Likert scale consisted of five points from 1 (“not at all”) to 5 (“very much so”), and the total score for each teacher level was calculated by averaging the summed scores for each participant. Reliability for this measure in this study was *α* = .912. In addition to the 15 questions evaluating course satisfaction, this survey contained three further questions with a nominal scale to ascertain whether participants were eligible for the study, i.e. yes/no questions on course completion and commencement or discontinuation of any other form of therapy or mental health treatment during the MBSR course, and a question on the number of teaching sessions attended out of a possible eight.

### Data Analyses

The L1 cohort, consisting of two teachers, each with one course participant taking part in the study, was too small to be representative and was therefore excluded from further analyses. Hence, the following analyses refer only to L2 and L3 results (teachers: L2 *n* = 4, 1 male, *M* age = 45; L3 *n* = 5, 2 males, *M* age = 52). After exclusions and non-completions detailed below under “Results”, 31 course participants who met all inclusion criteria remained in the analyses (L2 *n* = 9, 1 male, *M* age = 44; L3 *n* = 22, 5 males, *M* age = 47).

Several respondents did not complete some of the questionnaires: SCS-SF was not answered at all by one participant (from the L2 cohort); WBI-5 was missed out completely by another participant (from L3); PSS scale was fully omitted by three participants (two from L2 and one from L3). These participants were excluded from analysis of these questionnaires. FFMQ and Satisfaction Survey (SATF) were completed by all 31 qualifying course participants. Some questions from remaining respondents were left unanswered. Little’s Missing Completely at Random (MCAR) test was carried out to check for and compute missing values in pre- and post-test answers for each questionnaire (*p* > .05 for all missing values). All data were checked for normality of distribution, and dispersion and central tendency statistics were calculated to detect any outliers. One outlier was found in WBI-5 data for L3 and another in PSS outcomes for L3; these were excluded from further analyses. The means, standard deviations and gains (calculated by subtracting total scores before the MBSR training from the total scores after the MBSR course) are summarised in Table 2.

**Table 2:** Individual means (SDs) for mindfulness and well-being observations of L2 and L3 groups

Measure	L2 group				L3 group			
	*n*	Pre	Post	Gains	*n*	Pre	Post	Gains
		(SD)	(SD)	(SD)		(SD)	(SD)	(SD)
FFMQ	9	121.33	140.11	18.78	22	112.77	139.14	26.36
		(21.21)	(26.04)	(24.4)		(17.78)	(15.78)	(11.06)
SCS-SF	8	38.38	45.00	6.63	22	31.55	41.09	9.55
		(12.49)	(7.01)	(6.91)		(5.91)	(6.14)	(5.63)
WBI-5	9	15.67	15.11	−.56	20	12.80	16.15	3.35
		(4.85)	(4.94)	(6.63)		(4.71)	(3.92)	(3.30)
PSS	7	17.00	15.86	−1.14	20	20.60	14.40	−6.46
		(8.27)	(6.01)	(10.57)		(5.84)	(4.46)	(5.58)
SATF	9		56.11		22		64.64	
			(10.63)				(7.02)	

*FFMQ* Five Facet Mindfulness Questionnaire, *SCS-SF* Self-Compassion Scale—Short Form, *WBI-5* WHO (Five) Well-being Inventory, *PSS* Perceived Stress Scale, *SATF* Satisfaction Survey

The main analyses compared outcomes of MBSR course participant questionnaires between the two teacher groups. To this aim, two-way mixed analyses of variance (ANOVAs) were conducted for each of the standardised measures with factors of group (L2, L3) and time (T1: before MBSR, T2: after MBSR). Any significant interactions were further investigated for predicted directionality of differences with *t* tests. Additional analyses looked into other teacher predictor variables that might relate to participant outcomes, specifically age and experience in mindfulness-based teaching, meditation and retreat.

## Results

### Exclusions and Attrition

From the L2 cohort, one course participant needed to be excluded because of discontinuing the course. This was the only person from the original 52 volunteering for this feasibility pilot study where course attrition was confirmed, resulting in a known retention rate of course participants of 98%. Another participant from L2 was excluded because they failed to provide information on whether they had started or discontinued another form of mental health treatment. In addition to these two exclusions, two other course participants were in the non-completers category because they did not complete both pre- and post-test questionnaires. From the original 37 course participants in the L3 cohort volunteering for the study, two were excluded because they had started or discontinued another mental health treatment. A further 13 from this cohort were non-completers, of which three only completed the post-test questionnaire and several of the remaining non-completers were likely impacted by technical difficulties in accessing the online link to questionnaires, even though not all of them reported such difficulties to the researcher. This meant the overall drop-out rate for both cohorts due to non-completion was 30%. Exclusions and attrition are summarised in Table 3.

**Table 3:** Overview of exclusions and attrition of MBSR course participants in study, in L2 and L3 groups

	L2 Group	L3 Group
	*n*	*n*
Original volunteers for study	13	37
Attrition (course not completed)	1	–
Exclusions due to:		
Changes in other treatment	–	2
No information on possible changes in other treatment	1	–
Non-completion of questionnaires	2	13
Remaining MBSR course participants in study	9	22

*L2 Group* cohort of courses taught by teachers with 2 years of teacher training, *L3 Group* cohort of courses taught by teachers with 3 years of teacher training

The directional hypothesis that MBSR participants taught by teachers with higher levels of training would achieve greater gains was tested for each of the measures. To ensure analysis, results were not skewered by baseline differences between the two groups and independent between-group *t* tests were done for all four mindfulness and well-being measures, comparing L2 and L3 groups at a pre-test baseline level. Results for the respective measures were FFMQ *t*(29) = 1.51, *p* = .26; SCS-SF *t*(28) = 1.49, *p* = .17; WBI-5 *t*(27) = 1.50, *p* = .14; and PSS *t*(25) = −1.26, *p* = .22, indicating no significant differences at baseline between the two groups.

The 2 × 2 ANOVA for mindfulness measured by using FFMQ showed no main effect of group (*F*(1,29) = .49, *p* = .49, *ŋ* ^2^ = .02), but there was a significant main effect of time (*F*(1,29) = 51.45, *p* < .001, *ŋ* ^2^ = .64). There was no significant interaction (*F*(1,29) = 1.45, *p* = .24, *ŋ* ^2^ = .05).

The 2 × 2 ANOVA for the self-compassion measures, SCS-SF, revealed a marginally significant effect of group (*F*(1,28) = 3.85, *p* = .06, *ŋ* ^2^ = .12) and a significant effect of time (*F*(1,28) = 42.98, *p* < .001, *ŋ* ^2^ = .61). However, there was no significant interaction (*F*(1,28) = 1.40, *p* = .25, *ŋ* ^2^ = .05).

**Fig. 1:**
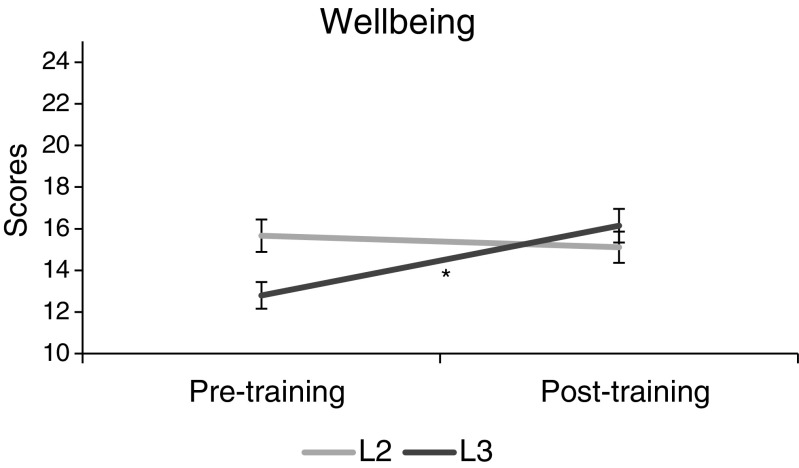
Well-being scores (WBI-5) change from before to after MBSR training, indicating a non-significant change in the L2 group and a highly significant increase (*p* < .001) in the L3 group (95% CI)

The 2 × 2 ANOVA for WBI-5 showed no main effect for group *F*(1,27) = .34, *p* = .56, *ŋ* ^2^ = .01) or time *F*(1,27) = 2.34, *p* = .14, *ŋ* ^2^ = .08), but there was a significant interaction (*F*(1,27) = 4.57, *p* = .04, *ŋ* ^2^ = .14). Follow-up *t* tests did not reveal any significant pre-post changes in the L2 group (*t*(8) = .25, *p* = .81, *d* = .08), but the pre-post comparisons in the L3 group were highly significant with large effect size (*t*(19) = −4.54, *p* < .001, *d* = −1.02) (Fig. 1).

**Fig. 2:**
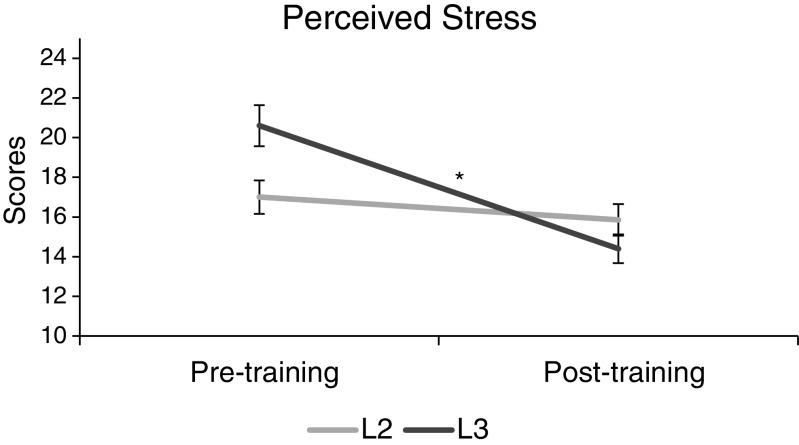
Perceived stress scores (PSS) change from before to after MBSR training, indicating a non-significant change in the L2 group and a highly significant decrease (*p* < .001) in the L3 group (95% CI)

The 2 × 2 ANOVA for PSS revealed no main effect of group (*F*(1,25) = .27, *p* = .61, *ŋ* ^2^ = .01), but there was a significant main effect of time (*F*(1,25) = 6.43, *p* = .02, *ŋ* ^2^ = .20), and there was a marginally significant interaction between group and time (*F*(1,25) = 3.05, *p* = .09, *ŋ* ^2^ = .11). Follow-up *t* tests did not show any significant change in the L2 group from pre to post (*t*(6) = .29, *p* = .78, *d* = .11), but the L3 group findings of pre-post comparisons were highly significant with a large effect size (*t*(19) = 5.93, *p* < .001, *d* = 1.33) (Fig. 2).

**Fig. 3:**
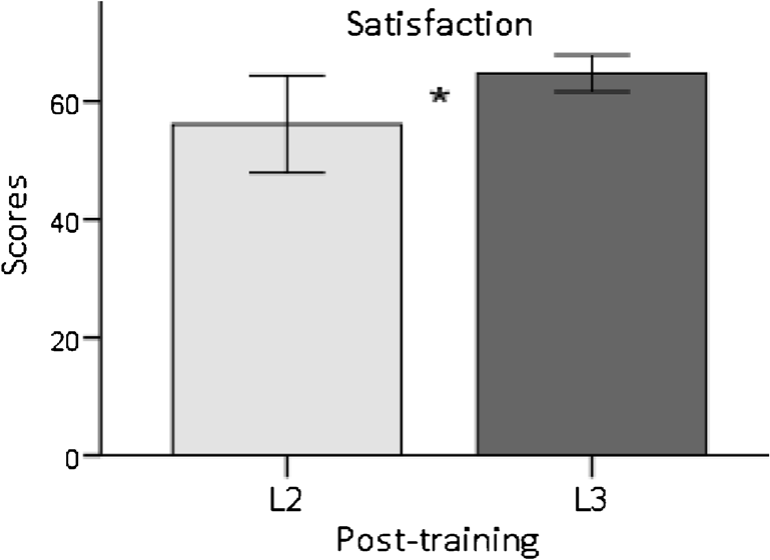
SATF Satisfaction Survey scores after MBSR training for L2 and L3, indicating a significant difference between the two groups (95% CI)

In order to assess significance of differences between course satisfaction scores for L2 and L3, an independent-samples *t* test was conducted, as this questionnaire could only be measured at the post-test level. The results showed a significant difference with large effect size (*t*(29) = −2.63, *p* = .01, *d* = −.95). The significant difference was due to a higher mean level of course satisfaction experienced by participants taught by teachers with a higher level of training (L3) (see Fig. 3).

Finally, an analysis was conducted on differences in course attendance amongst L2 and L3 course participants to check if this might have affected the difference in outcomes between the two groups of course participants. No significant differences between the groups were found on attendance levels (*t*(29) = −1.74, *p* = .11).

Of secondary interest was the relationship between participant outcomes and variables other than teacher training level, specifically meditation experience, age, number of courses taught, time spent practicing mindfulness in total and on average and time spent on retreat. Differences in teacher variables are demonstrated in Table 4, including previous clinical and/or educational experience or qualifications. Absence of specific information on clinical and educational experience and qualifications prohibited further analysis of differences between these variables.

**Table 4:** Demographics means for main teacher variables of L2 and L3 MBI teachers

	L2 teachers	L3 teachers
	*n* = 4 (1 male)	*n* = 5 (2 males)
Teacher variable	Mean (SD)	Mean (SD)
Age	45 (*8.8*)	52 (*8.5*)
Courses taught	18 (*12.7*)	44 (*34.5*)
Years of mindfulness	8 (*2.9*)	20 (*12.2*)
Days on retreat	48 (*31.1*)	176 (*159.7*)
Daily meditation in min	38 (*8.7*)	30 (*.00*)
Prior clinical background^a^	1	1
Prior educational background^a^	1	1
Prior clinical and educational background^a^	1	3

^a^Background refers to experience and/or qualifications

Wilcoxon rank-sum tests for differences in years of mindfulness practice and for number of days on retreat between the two teacher groups were marginally significant: years of mindfulness practice *z* = 1.96, *p* = .06; days on retreat *z* = 1.97, *p* = .06. No statistically significant differences were found between L2 and L3 for other teacher variables. To follow up on the marginally significant difference in years of practice and amount of retreat, the nine teachers were ranked in three groups, low, medium and high, according to their score for these two variables, creating three ranking groups of three teachers for each variable. Two-way mixed ANOVAs with factors of group (low, medium, high) and time (T1: before MBSR, T2: after MBSR) were conducted for the measures which showed significant between-group differences for L2 and L3 (WBI-5, PSS) and a one-way ANOVA for course satisfaction (SATF), with factor of group (low, medium, high), to see whether the years of mindfulness practice and amount of retreat could explain the differences observed. No significant or marginal interactions were obtained on any of the comparisons: ranking years of mindfulness practice: WBI-5 *p* = .85, PSS *p* = .66, SATF *p* = .72; ranking retreat time: WBI-5 *p* = .30, PSS *p* = .88, SATF *p* = .21.

## Discussion

This feasibility pilot study set out to investigate impact of MBSR/MBCT teacher training levels on well-being outcomes of MBSR course participants and hypothesised that higher level of teacher training would relate to greater well-being outcomes. We also explored relationships between teaching and meditation experience of teacher and participant outcomes. Specifically, this study focused on two groups of MBSR/MBCT teachers with different levels of postgraduate mindfulness-based teacher training and their respective MBSR course participants. Our findings revealed significantly better outcomes for well-being and significantly greater reductions in perceived stress for MBSR course participants taught by a teacher with a higher level of mindfulness-based teacher training. In addition, the analyses showed significantly higher satisfaction scores for the participant cohort taught by higher trained teachers. No significant differences were found between the two cohorts in score increases for the mindfulness and self-compassion measures. And contrary to our expectations, no evidence was found for a relationship between either MBSR/MBCT teachers’ teaching or meditation experience and participant outcomes.

The observed differences in participant outcomes between the two teacher levels might relate to incremental differences in training or to specific features of the higher training level. For example, at the higher training level, students are required to integrate a broader range of theoretical underpinnings into their essay and to link the theory to the specifics of the MBSR/MBCT curriculum and teaching process. This may provide qualitatively different insights to their teaching and thus strengthen their teaching. Another possibility is that cohorts of teachers progressing to this higher training level share characteristics which differentiate them from other teachers and which have not been captured in this study. In view of these preliminary findings, further investigation of possible mediating factors within teacher training in relation to optimum course participant outcomes is needed.

The study finding of greater well-being scores for participants taught by higher trained teachers raises the question: why was this difference not significant for mindfulness and self-compassion? Crane et al. (2012) offered a model for training-related developmental stages of MBP teachers, based on clinical application by Sharpless and Barber (2009) of pioneering work by Dreyfus and Dreyfus (1986) on development of competence. This clarified that there are distinct stages to skill acquisition, but there are many gaps in our understanding in the MBP teacher context regarding what skills develop at which stage of training. It is possible that most MBP teachers with a basic teacher training will be able to convey learning on mindfulness and self-compassion and that competencies related to instilling aspects of well-being develop more fully at later training stages. Another tentative explanation for difference in findings for mindfulness and self-compassion could be the specific use of Five Facet Mindfulness Questionnaire (FFMQ) and short form of Self-Compassion Scale (SCS-SF). These questionnaires have been subject to critique (Williams et al. 2014), in part because the concept of mindfulness may change during participation in an MBP. Hence, caution is recommended around the interpretation of the FFMQ and self-compassion results. There has been an ongoing debate about the construct and measurement of mindfulness, as alluded to earlier (Chiesa 2013; Grossman and Van Dam 2011). For this reason, further measures of well-being were used in this study, as recommended by Grossman (2008), as well as course satisfaction at the end of intervention.

The secondary intention of this study was to investigate whether teacher variables other than training level might impact on participant outcomes. Teacher demographics suggested that higher trained teachers differed significantly from lower trained counterparts, in aspects such as experience in MBP teaching and meditation, and retreat attendance. Interestingly, only mindfulness-based teacher training and no other teacher variables resulted in significant participant outcome differences. The non-significant results included the amount of retreat experience by teachers. Time spent on retreat might be connected to development of embodiment, considered an instrumental factor for teachers (Grossman 2015) and one of the domains of the MBI-TAC. The contrast with significant findings related to teacher training is notable. In addition, no significant differences were found in terms of course attendance, suggesting this aspect of MBSR training did not have influence on differences in outcomes either, thus strengthening the hypothesis of mindfulness-based teacher training as a possible strong predictor of participant outcomes.

Besides informing mindfulness-based teacher training, findings of this study could have other practical implications for MBP teaching. Psychiatrists have warned against underqualified teachers offering MBPs within the health service (Booth 2014), and Hyland (2015) cautioned against use within industry of “Short-term McMindfulness strategies [to] offer quick-fix solutions” (p. 231). The challenge of MBP implementation has been highlighted by Dimidjian and Segal (2015) and in the recent report by the UK Mindfulness All-Party Parliamentary Group (MAPPG) (2015). Evidence speaking to the issue of teacher training is needed both to support the developing science of MBPs (Dimidjian and Segal 2015) and the implementation challenge (Rycroft-Malone et al. 2014). The level of teacher training of clinicians offering MBPs is rarely mentioned in research studies yet based on our findings could make a significant difference to outcomes. Awareness of this could encourage researchers to include information on training levels of study clinicians and allow more equitable evaluations of MBP studies. This information could also assist the general public in choosing mindfulness-based courses taught by adequately trained teachers and benefitting accordingly from the integrity of the program.

### Strengths and Limitations

This study has a number of strengths. Firstly, it begins to address the need for further investigation into MBP teacher impact as recognised by Khoury et al. (2013), Dimidjian and Segal (2015) and Van Aalderen et al. (2014). It expands on qualitative research by the latter on the role of MBCT teachers in conveying mindfulness and in the therapeutic relationship with course participants. The current feasibility pilot study is an initial investigation measuring impact of mindfulness-based teacher training on participant outcomes, addressing the need for further investigation of MBP clinician training (Dimidjian and Segal 2015, p.605) within the context of stage I of the six-stage NIH model for intervention research (Onken et al. 2014). Stage I research is concerned with the creation and refinement of a new intervention in this case MBPs. In their mapping of the MBP evidence base, Dimidjian and Segal (2015) also found that whilst a large body of literature exists in the first three NIH developmental stages of MBP evidence, there is a scarcity of research into the later three stages. It can be argued that this feasibility pilot study has significant relevance for these later research stages, namely efficacy in community trials (stage III), effectiveness research (stage IV) and implementation and dissemination studies (stage V), which are critical to the successful implementation of MBPs. The findings of this feasibility pilot study can help to inform further investigation of these complex issues. Secondly, the finding of significant differences in well-being and stress outcomes between training cohorts supports the assumed importance of teacher training, particularly since no similar result was found for other teacher variables. Furthermore, statistically significant differences in longitudinal outcomes were coupled with large effect sizes, a combination considered as “single best estimate” of divergence from null hypothesis (Fritz et al. 2012, p. 104).

A number of important limitations need to be considered. First, the cohort with the lowest level of teacher training had to be excluded from analysis because of low teacher and participant numbers, and data analyses were conducted only on the teacher groups with the higher and highest training levels. This limits the strength of the findings; inclusion of the lowest training level would have possibly further highlighted differences between the highest and lowest levels of training. However, the difficulty in recruiting the group with the lowest level of teacher training, possibly explained by a lack of confidence in their own teaching abilities, also provided useful feasibility guidance for further larger scale studies in terms of recruitment challenges and need to maximise recruitment intake. This could be investigated further in qualitative research, with a view to identifying the facilitators and barriers to their participation, and based on this, ensuring this group is included in future studies comparing groups of teachers with different levels of teacher training. More specific information on clinical and educational experience/qualifications of teachers could also be obtained to investigate whether and if so how such mindfulness non-specific differences might impact on participant outcomes. Secondly, whilst a relatively small sample size can be expected for a pilot study in a naturalistic setting, since study participants took part on a voluntary basis and were recruited from real-world courses (not research courses), overall sample size was small and sample sizes between cohorts varied considerably due to the larger number of courses taught by teachers with the highest training levels. Additionally, the proportion of volunteers who did not complete both pre- and post-test questionnaires varied noticeably between cohorts, probably to a large extent due to problems accessing online questionnaires. Therefore, the results should be interpreted with caution. A mitigating factor for our findings is that the average percentage of participating course participants per course was very similar for the two cohorts which we compared. These constraints and challenges also serve as useful discoveries in terms of the challenge of researching the research questions this study aimed to address. It is imperative to follow up these initial findings with further research, particularly larger studies, into MBP teacher impact, and to build on the learnings from this feasibility pilot study for optimising the number of study participants, selection of relevant measures and teacher variables.

Finally, this study was conducted with teachers from one training institution, and findings may not be easily transferable to teachers trained elsewhere. Further investigations into relationships between teacher training and participant outcomes would advance our understanding, particularly if they include and compare teacher populations with both academic and non-academic training routes, and with MBP courses other than MBSR, e.g. MBCT and teacher-led low dose courses such as MBSR-ld (Hülsheger et al. 2015; Klatt et al. 2009). Study design of future studies could also be expanded to include measurement of teaching competence, by combining outcomes with MBI-TAC scores of participating teachers and through investigating relationships between training levels, competence and outcomes.

In conclusion, the primary findings showed that participants taught by higher trained teachers had significantly greater outcomes on well-being and reductions in perceived stress, but not on mindfulness and self-compassion. The second major finding was that no effect of other teacher variables on participant outcomes, including meditation and retreat experience, was found. Taken together, these results support the hypothesis that higher teacher training may be related to greater well-being outcomes of course participants. Despite its limitations due to small and variable group sample sizes and non-randomised nature of the design, this study is an important initial step in investigating links between mindfulness-based teacher training and participant outcomes, with implications for mindfulness-based teaching and implementation of MBPs in clinical and non-clinical contexts.
